# Associations of maternal inflammatory states with human milk composition in mothers of preterm infants

**DOI:** 10.3389/fnut.2023.1290690

**Published:** 2024-02-02

**Authors:** Erin Landau-Crangle, Deborah O’Connor, Sharon Unger, Kathryn Hopperton, Emily Somerset, Hadar Nir, Rebecca Hoban

**Affiliations:** ^1^The Hospital for Sick Children, Toronto, ON, Canada; ^2^Department of Pediatrics, University of Toronto, Toronto, ON, Canada; ^3^Sinai Health System, Toronto, ON, Canada; ^4^Ted Rogers Centre for Heart Research, The Hospital for Sick Children, University Health Network, Toronto, ON, Canada; ^5^Department of Pediatrics, University of Washington, Seattle, WA, United States; ^6^Seattle Children’s Hospital, Seattle, WA, United States

**Keywords:** human milk, human milk cytokines, preterm infant, maternal obesity, maternal inflammation during pregnancy, human milk composition, pre-eclampsia

## Abstract

**Introduction:**

Overweight/obesity (ow/ob) is increasing in prevalence in pregnant women, and it is associated with other pro-inflammatory states, such as pre-eclampsia, gestational diabetes, and preterm labor. Data are lacking if mothers experiencing inflammatory states who deliver preterm have mother’s own milk (MOM) with differing inflammatory markers or pro-inflammatory fatty acid (FA) profiles.

**Methods:**

The aim was to explore associations of maternal pre- and perinatal inflammatory states with levels of inflammatory markers and/or FAs in longitudinal samples of MOM from mothers of preterm infants born <1,250 g. Inflammatory states included pre-pregnancy ow/ob, diabetes, chorioamnionitis (chorio), preterm labor (PTL), premature rupture of membranes (PROM), pre-eclampsia, and cesarian delivery. In MOM, inflammatory markers studied included c-reactive protein (CRP), free choline, IFN-Ɣ, IL-10, IL-1β, IL-1ra, IL-6, IL-8, and TNF-α, and FAs included omega-6:omega-3 ratio, arachidonic acid, docosahexaenoic acid, linoleic acid, monounsaturated FAs, and saturated FAs. The above inflammatory states were assessed individually, and the healthiest mothers (normal BMI, no chorio, and ± no pre-eclampsia) were grouped. Regression analysis tested associations at baseline (day 5) and over time using generalized estimating equations.

**Results:**

A total of 92 infants were included who were delivered to mothers (42% ow/ob) at a median gestational age of 27.7 weeks and birth weight of 850 g. MOM CRP was 116% higher (relative change 2.16) in mothers with ow/ob at baseline than others (*p* = 0.01), and lower (relative change 0.46, 0.33, respectively) in mothers in the two “healthy groups” at baseline (both *p* < 0.05) than others. MOM IL-8 levels were lower with chorio and PTL at baseline. No significant associations were found for other individual or grouped inflammatory states nor for other MOM inflammatory markers nor FA profiles at baseline.

**Discussion:**

In conclusion, MOM CRP levels are positively associated with inflammatory states, such as ow/ob. Reassuringly, there was no association between FA profiles or most other inflammatory markers and maternal inflammatory states. Further studies are needed to determine potential associations or ramifications of MOM CRP in vulnerable preterm infants.

## Introduction

The prevalence of pre- and peri-pregnancy overweight/obesity (ow/ob) has risen substantially in the last 40 years, and it is associated with co-morbidities affecting both mother and infant, including gestational diabetes, hypertension, pre-eclampsia, and higher rates of premature rupture of membranes (PROM), preterm labor (PTL), and cesarian delivery (1, 2). This increase has significant societal justice and resource utilization ramifications as ow/ob disproportionately affects racialized women and, therefore, their vulnerable infants (2) and is associated with an approximately 30% higher rate of preterm delivery than healthy-weight women (3). This difference may, in part, be explained by higher levels of inflammation (4) as ow/ob is a pro-inflammatory state marked by elevated serum c-reactive protein (CRP) levels (5–8). Higher levels of inflammation in pregnancy may lead to increased insulin resistance and gestational diabetes (7), with associated maternal and neonatal complications. In addition, pre- and peri-pregnancy pro-inflammatory states like ow/ob are often co-morbid with others, including surgical/cesarian delivery, pre-eclampsia, and chorioamnionitis (chorio), which are known to influence fetal metabolic programming and may impact neurodevelopment and increase risks of childhood obesity (7). It is unclear whether there may be a cumulative effect of multiple pro-inflammatory “hits” on the developing fetus (7, 9) and subsequent infant outcomes, such as whether such early-life programming may continue during lactation.

Mother’s own milk (MOM) is the gold standard for feeding preterm infants, improving neurodevelopment and lowering rates of co-morbidities, including necrotizing enterocolitis (10). Like many other biomolecules, cytokines from the maternal serum, or potentially the mammary gland, pass into MOM. MOM cytokines and their potential role or effect on the infant are a relatively new and increasing focus of human milk research. Data are lacking on whether mothers experiencing inflammatory states, including ow/ob, who deliver preterm infants have MOM with altered profiles of inflammatory markers compared to mothers without or with fewer inflammatory states. Studies have shown pro-inflammatory fatty acid (FA) profiles, with higher ratios of omega-6 to omega-3 s, in MOM of mothers of term infants who had pre-pregnancy and peri-pregnancy ow/ob than MOM from normal weight mothers, with optimization potentially possible with dietary interventions (11, 12).

The objective of this study was to investigate associations, if any, of maternal inflammatory states with subsequent levels of inflammatory markers and FAs in MOM from mothers of preterm infants. Inflammatory states studied included pre-pregnancy ow/ob, diabetes, chorio, PTL, PROM, pre-eclampsia, and caesarian delivery. Inflammatory markers included cytokines, CRP, and free choline, which have been reported to inversely correlate with CRP (13, 14). FAs studied included omega-6:omega-3 ratio, docosahexaenoic acid (DHA), arachidonic acid (ARA), linoleic acid, monounsaturated FAs, and saturated FAs. This was a preplanned analysis of a larger exploratory study (15) describing longitudinal cytokine and other inflammatory marker profiles in MOM from mothers of preterm infants.

## Materials and methods

### Study site and participants

This study is an exploratory secondary analysis of data and MOM samples from a prospective, triple-blind, randomized controlled trial (16) (OptiMoM; NCT02137473) of 127 very low birth weight (VLBW) infants and their mothers. Infants were eligible if birth weight was <1,250 g and were feeding MOM and/or consented to supplemental donor milk. Exclusion criteria included receipt of formula or fortifier before enrollment, no feeds within 14 days of birth, and congenital anomalies affecting growth. Enrollment in the original study occurred at the Hospital for Sick Children and Mount Sinai Hospital, two-level III NICUs, in Toronto, Canada, in 2014–2015, an ethnically diverse city where half of the population are visible minorities and/or immigrants (17). Mothers provided signed informed consent for themselves and their infants. Mothers previously enrolled in the OptiMoM study who still had frozen MOM sample(s) available that were collected at the end of each postpartum week (±2 days) from each of postpartum weeks 1–4, 7–8, or 10–11 were eligible for this secondary study. Mothers of singletons and multiples were included, with the first infant of multiples enrolled in the original study utilized for infant-level data. As the exploratory analysis required data from the electronic medical record (EMR) that had not been originally collected (maternal pre-eclampsia, PTL, PROM, diabetes, and infant diet at NICU discharge), eligible mothers were re-consented via phone to allow access to the EMR to obtain these data and to use frozen MOM samples for testing outside the original consent of “nutritional components.” Mothers whose infants died during the original study were not reapproached, nor was contact attempted for dyads known to have been lost to follow-up. In these few cases, only data that were available from the original study were utilized (mother/infant medical/sociodemographic data, MOM FAs). MOM samples were only retested for components that fell within the nutritional realm (free choline). In all eligible mothers, frozen MOM samples that were subject to secondary analysis were matched (ideally another aliquot of the same sample with the same time/date, or if not available, then within 2 days) to MOM samples that had been previously analyzed for FAs. Longitudinally collected MOM samples were analyzed for cytokines and inflammatory markers (CRP, free choline, IFN-Ɣ, IL-10, IL-1β, IL-1ra, IL-6, IL-8, and TNF-α) and FA composition (omega-6:omega-3 ratio, percentage of ARA, DHA, linoleic acid, monounsaturated FAs, and saturated FAs) at baseline and over time. The above inflammatory states were assessed separately, and mothers, with and without such states, were grouped for comparisons *a priori* to better understand the effect of multiple inflammatory morbidities (or lack thereof). To group “healthier” mothers, we first excluded mothers with ow/ob as this diagnosis is well known to be associated with long-term inflammation (4–8). With a relatively small “n” and high prevalence of ow/ob in the population, we then stepwise added the two short-term inflammatory diagnoses in our database that are more definitively associated with inflammation (chorio and pre-eclampsia as PTL, for example, may or may not be associated with inflammation depending on the etiology, such as infection vs. incompetent cervix). Mothers with inflammatory states were then compared to healthy group 1 (normal BMI, no chorio) and healthy group 2 (normal BMI, no chorio, and no pre-eclampsia). Both the original trial and this exploratory analysis were approved by both hospitals’ Research Ethics Boards.

### Assessment of milk inflammatory markers

All milk analyses aside from FA testing were performed by the Analytical Facility for Bioactive Molecules, The Hospital for Sick Children, Toronto, Canada. Milk samples were thawed and centrifuged to remove the top fat layer. The skimmed product was separated into aliquots and used in duplicate for all analyses. A custom cytokine multiplex bead panel assay was performed for IL-1βeta, IL-1ra, IL-6, IL-8, IL-10, IFN-gamma, and TNF-alpha (HCYTOMAG-60 K-07 human cytokine magnetic kit; Millepore Sigma). Detection limits were 1.09, 0.95, 1.90, 1.69, 0.81, 0.66, and 1.00 pg./mL, respectively. CRP was quantified with a magnetic bead multiplex assay (HNDG2MAG-36 K-01.Neurodegenerative MAG Panel 2; Millipore Sigma) using assay buffer; samples were run at 1:100 dilution with a detection limit of 0.003 pg./mL. Sample data for cytokine and CRP were processed with Milliplex Analyte version 5.1.0.0. Free choline was quantified using a choline colorimetric enzyme assay kit (MAK056; Sigma-Aldrich) with a detection limit of 0.01 nmol/μL. Sample results in nmol/μL were converted to ng/μL by multiplying by the molecular weight of choline (104.2 ng/nmol).

FA analyses on aforementioned paired unskimmed milk samples had previously been performed via gas chromatography and described (18), with each subtype reported as the percentage of total FAs. The omega-6:omega-3 ratio was calculated from respective percentages of omega-6 and omega-3 FAs.

### Statistical analysis

Baseline clinical characteristics were summarized using descriptive statistics. Continuous variables were summarized as means and standard deviations or medians and interquartile ranges (IQRs), and dichotomous and polytomous variables were summarized as frequencies.

To visualize the potentially nonlinear time profile of serial MOM inflammatory markers (log-transformed) and FA profiles, in keeping with our previous studies, we modeled the time effect using natural cubic splines and generalized estimating equations (GEEs) with an identity link function and independent working correlation matrix. The corresponding pointwise 95% confidence intervals (CIs) were estimated using a robust sandwich estimator (15). Cytokines, CRP, and free choline were log-transformed; FAs were reported as percentages in the original data; FA values were multiplied by 100 to compensate for the large differences in units.

We assessed and quantified the association of maternal inflammatory states with MOM inflammatory markers and FA profiles using GEEs with identity link functions and an independent work correlation matrix. Due to the lack of MOM sample availability before day 4 and low numbers of samples in weeks 10–11 (less than 10% of subjects contributed samples in this time period), we analyzed all MOM samples from day 5 (henceforth, referred to as baseline) to day 54. Sample collection time (days) was considered a covariate and modeled using natural cubic splines. Specifically, we investigated the interaction between time and maternal inflammatory states and assessed if any maternal inflammatory states altered the time profiles of the milk inflammatory markers or FA profiles. The corresponding pointwise 95% CIs and *p*-values were estimated using a robust sandwich estimator.

Finally, we quantified the associations between FAs and inflammatory markers in the cohort overall as well as “healthy group 1” and “not healthy” (aka not in “healthy group 1”). Each association was adjusted for a time trend and was estimated using GEE with identity link functions and an independent working correlation matrix.

It is noteworthy that considerable proportions of serial observations were left censored (i.e., below the detection limit of the kits; see Supplementary Table S1, for missingness at each time point). We used a censored likelihood imputation method to account for these partial observations (19). Specifically, we imputed the left-censored observations 15 times each and estimated the final model using Rubin’s rule. Concurrent values of FAs were used as predictors in the imputation model. All statistical analyses assumed a statistical significance of 5% and were conducted using R v3.5.3.

## Results

**Figure 1:**
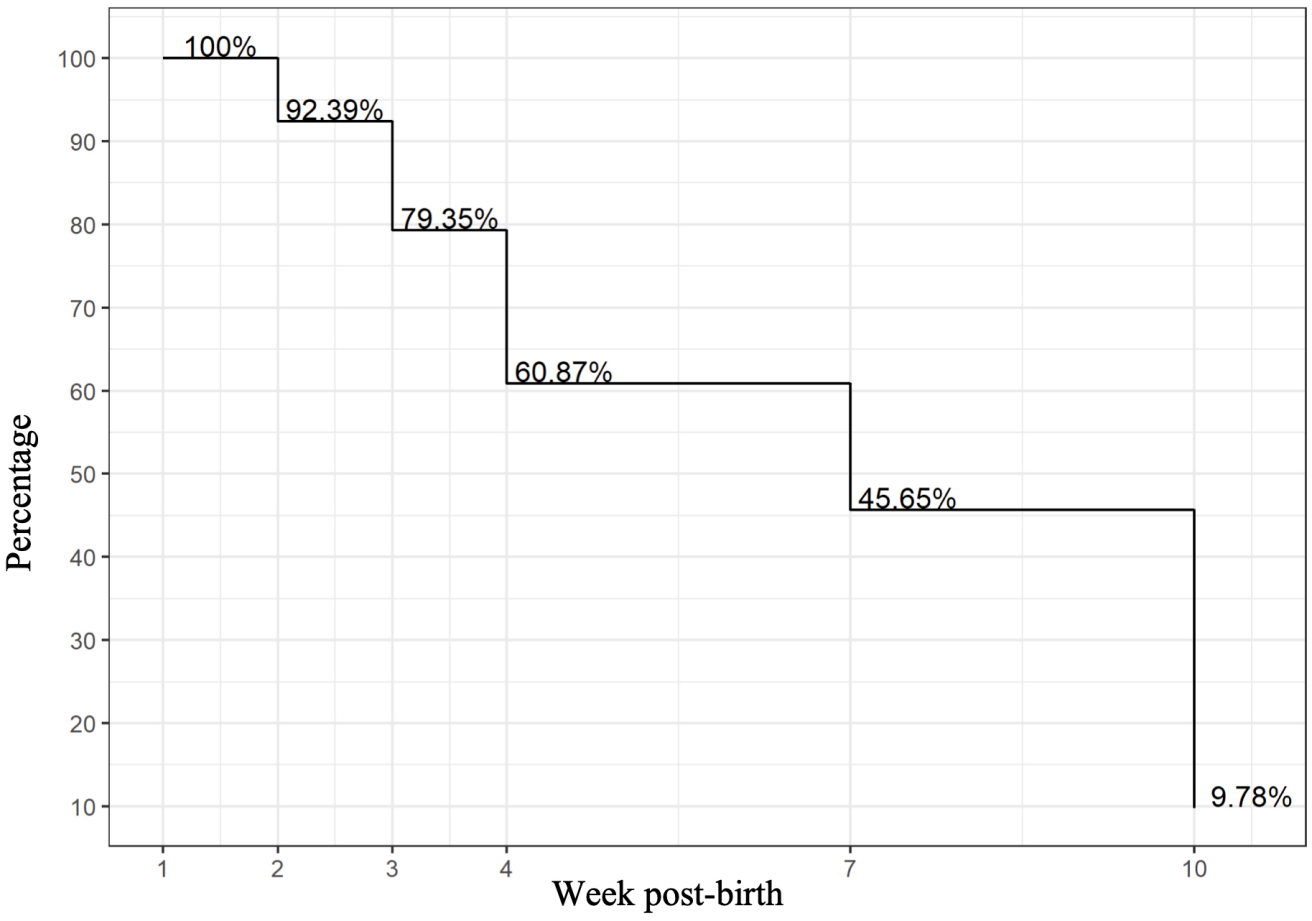
Percentage of infant-mother dyads with milk samples available for analysis by week.

Of the 127 VLBW infant–mother dyads in the OptiMoM study, 92 infant–mother dyads had MOM samples that met inclusion criteria (Supplementary Figure S1). Reflecting Toronto’s diversity, over two-thirds of the OptiMoM study cohort consisted of infant–mother dyads who were visible minorities, with the majority East or South Asian (16). A total of 226 MOM samples were analyzed and grouped into six time points: weeks 1, 2, 3, 4, 7–8, and 10–11. Inflammatory marker levels over time have been previously reported for the cohort in general (15). Subject characteristics are shown in Table 1—maternal co-morbidities and pro-inflammatory states were frequent, with a mean of 2.3 and a maximum of 5 inflammatory diagnoses per mother; every mother carried at least one inflammatory diagnosis. Ow/ob was very common; when this diagnosis was excluded, mothers had a mean of 2.0 additional diagnoses. Mother–infant dyads with remaining samples at each time point are illustrated in Figure 1. In this large exploratory analysis of cytokines, we found few associations, so given the large amount of data analyzed, we report and show figures only those that were statistically significant with potential clinical relevance (for full results, see Supplementary Table S2).

**Table 1:** Subject characteristics.

N=92		Median [IQR] or n (%)		
		Entire Cohort	Non-overweight/obese (*n* = 53)	Overweight/obese (*n* = 39)
Infant gestational age at birth (weeks)		27.7 [25.6, 29.6]	27.4 [26.3, 29.9]	27.7 [25.4, 29.1]
Infant birth weight (grams)		850 [735, 1035]	910 [740, 1070]	800 [710, 930]
Male infant		37 (45%)	20 (41%)	17 (52%)
Exclusive MOM feeds during NICU admission		46 (49%)	26 (49%)	20 (53%)
Maternal age (years)		33 [31, 37]	33 [31, 37]	33 [29, 38]
Pre-pregnancy BMI (kg/m^2^) category			
Normal/underweight (<25)		53 (58%)	53 (100%)	–
Overweight (25 – <30)		12 (13%)	–	12 (30.8%)
Obese (≥30)		27 (29%)	–	27 (69.2%)
Diabetes		4 (5%)	2 (4%)	2 (6%)
Cesarian delivery		57 (61%)	32 (60%)	24 (63%)
Chorioamnionitis		31 (38%)	20 (43%)	11 (31%)
Pre-eclampsia		20 (24%)	11 (22%)	9 (27%)
Preterm labor		41 (49%)	26 (53%)	14 (42%)
Premature rupture of membranes		26 (32%)	18 (37%)	8 (25%)

MOM: mother’s own milk; NICU: neonatal intensive care unit; BMI: body mass index.

Note: some characteristics don’t add up to N=92 due to missing data.

**Figure 2:**
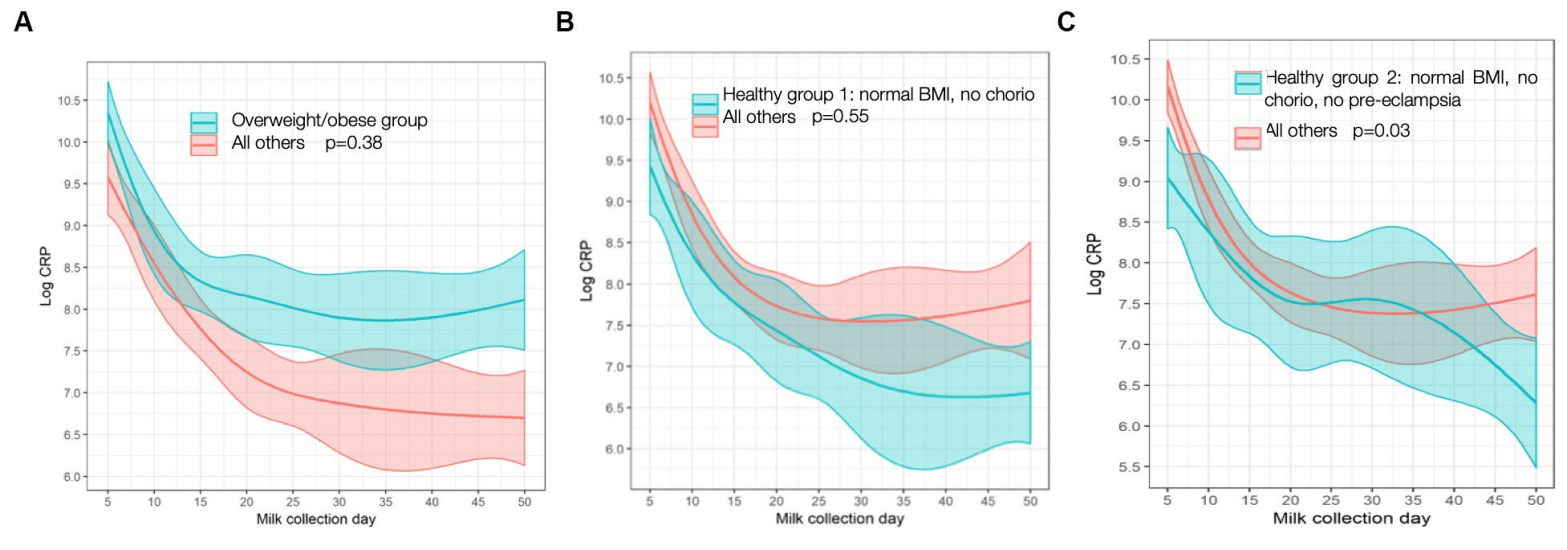
C-reactive protein (CRP) trajectory in: **(A)** Overweight/obese vs. all others; **(B)** “Healthy group l” vs. all others; **(C)** “Healthy group 2” vs. all others. *p* values are for differences in trajectory over time. BMI, body mass index; chorio, chorioamionitis.

In the two “healthy” groups, “healthy group 1” (normal BMI and no chorioamnionitis) consisted of *n* = 33 mothers. “Healthy group 2” (normal BMI, no chorio, and no pre-eclampsia) consisted of *n* = 23 mothers. MOM CRP concentrations were 116% higher (odds ratio, interquartile range [OR, IQR] 2.16 [1.21, 3.88]; Table 2) in ow/ob mothers at baseline than others and remained higher throughout the study (Figure 2). CRP concentrations were lower in mothers in the two “healthy groups” at the day 5 baseline than in the rest of the cohort, as seen in Table 2. In all groups, CRP decreased over time (Figure 2); similar rates of change were seen between ow/ob and healthy groups (*p* = 0.38). CRP rates of change over time only differed in “healthy group 2” compared to the rest of the cohort (Figure 2), where CRP concentrations decreased continuously over time instead of plateauing after an initial decrease in the rest of the cohort. No significant associations with other maternal inflammatory states were noted with CRP concentrations at baseline, nor differences in rates of change over time.

**Table 2:** Regression results for c-reactive protein.

N=92; *n* = 226 MOM samples	Relative Change [95% CI] at day 5 compared to entire cohort	*p* value
Overweight/obese *n* = 39 (42%)	2.16 [1.21, 3.88]	0.01
Healthy Group 1: normal BMI, no chorio *n* = 33 (36%)	0.46 [0.23, 0.91]	0.03
Healthy Group 2: normal BMI, no chorio, no pre-eclampsia *n* = 23 (25%)	0.33 [0.16, 0.66]	<0.01

MOM: mother’s own milk; BMI: body mass index; chorio: chorioamnionitis.

**Figure 3:**
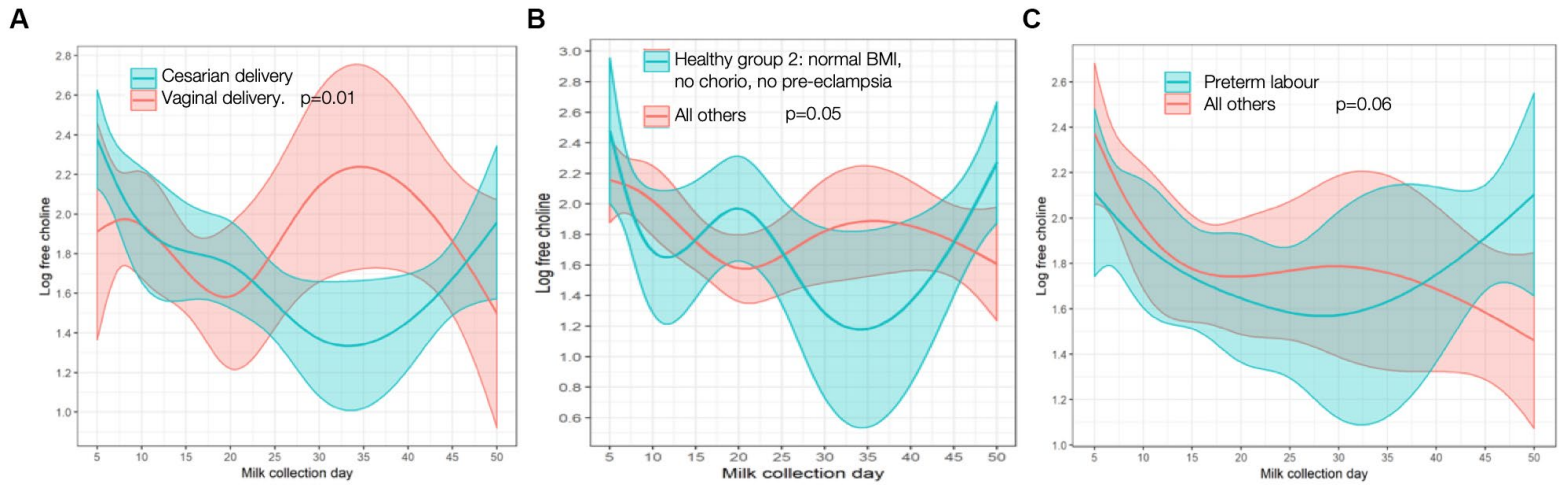
Free choline trajectory in **(A)** cesarian vs. vaginal delivery; **(B)** “Healthy group 2” vs. all others; **(C)** preterm labor vs. all others. *P-*values are for differences in trajectory over time. BMI, body mass index; chorio, chorioamionitis.

MOM IL-8 levels, an inflammatory cytokine, were lower in mothers with chorio (relative change [95% CI] 69.4% [8.06, 89.9%], *p* = 0.04) and PTL (relative change [95% CI] 63.3% [2.08, 86.2%], *p* = 0.05) at baseline than mothers without these diagnoses; trajectories over time did not differ. No other cytokines had any significant associations with maternal inflammatory states. Although there were no baseline differences, free choline demonstrated a significantly different time trend/shape in mothers who had cesarian sections (*p* = 0.01) compared to vaginal deliveries. As seen in Figure 3, free-choline levels decrease for the first month and then increase in mothers with cesarian sections; an opposite trajectory is seen in mothers who deliver vaginally. Differences in free choline trajectories were also seen in the “healthy group 2” (*p* = 0.05) compared to the rest of the cohort, and they had a borderline different trajectory in those with PTL (*p* = 0.06) compared to others (Figure 3). No significant associations were found for other individual or grouped inflammatory states for other MOM inflammatory markers at baseline (day 5), nor did trajectories differ over time when compared to the cohort as a whole.

**Figure 4:**
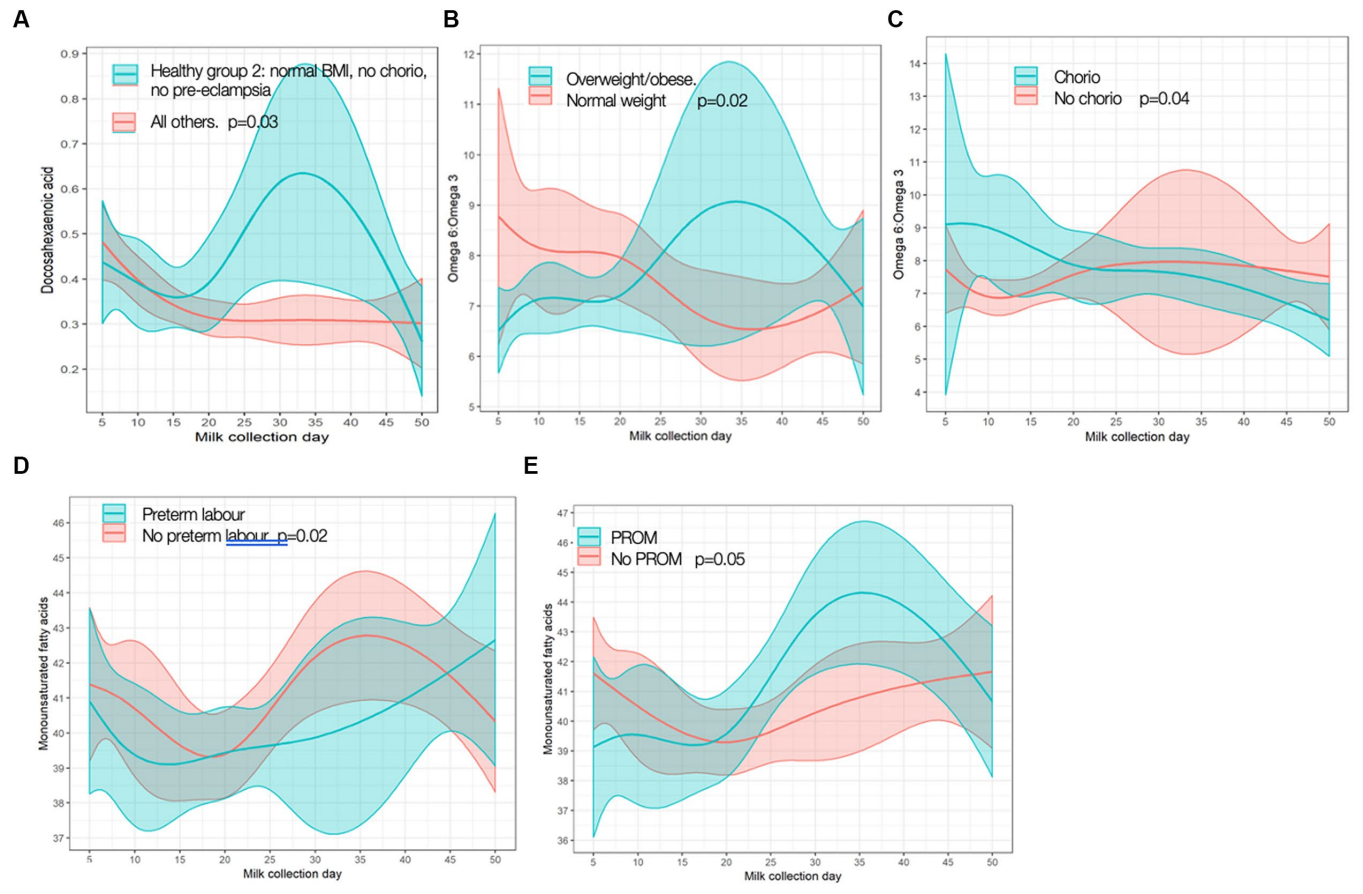
Significant differences in fatty acid trajectories. **(A)** Docosahexamoic acid (DHA) in Healthy Group 2 vs. all others. **(B)** Omega-f; pmggg-3 ratio over time in overweight/obese vs. normal weight. **(C)** Omega-qpmggg-3 ratio over time in chorioamnionitis (chorio) vs. no chorio. **(D)** Monounsaturated fatty acid (MUFA) over time in preterm labor (PTL) vs. no PTL. **(E)** MUFA over time in premature rupture of membranes (PROM) vs. no PROM p values are for differences in frajectory over time.

In exploring FAs in relation to maternal inflammatory states, we found few associations; again, only statistically significant results are shown given copious data (for full results, see Supplementary Table S2). “Healthy group 1” had lower levels of saturated FAs at baseline (change −4.24, [95% CI −7.71, −0.773], *p* = 0.02), and mothers with chorio had higher baseline levels of saturated FA (change 4.06 [0.149, 7.98], *p* = 0.04) when compared to others. “Healthy group 2” had different trajectories for DHA content compared to the cohort, with healthy mothers having higher MOM DHA levels over time than those not in “healthy group 2” (Figure 4A). Obese/overweight mothers had different trajectories for omega-6:omega-3 ratio than normal weight mothers (Figure 4B), as well as a trend toward lower monounsaturated FAs (MUFA) levels at baseline (change −3.38 [−6.91, 0.152], *p* = 0.06). Differences in omega-6:omega-3 trajectory with chorio vs. no chorio were also observed (Figure 4C). MUFA trajectory differences were seen with PTL and PROM (Figures 4D,E, respectively) compared to the rest of the cohort.

Finally, we explored associations between MOM inflammatory markers and FAs (Table 3). Given a plethora of data, only statistically significant associations are shown, focusing on markers reported in earlier sections (such as CRP and IL-8; for full results, see Supplementary Table S3).

**Table 3:** Associations between mother’s own milk inflammatory markers and fatty acids.

Predictor variable	Response variable	Coefficient [95% CI]	*p*
Log CRP	DHA	Overall: −0.023 [−0.046, 0.001]	0.056
		Healthy: −0.031 [−0.06, −0.002]	0.03
		Non-healthy: −0.005 [−0.037, 0.026]	0.74
	Omega-6	Overall: 0.247 [−0.173, 0.667]	0.25
		Healthy: 0.752 [0.053, 1.45]	0.04
		Non-healthy: 0.012 [−0.506, 0.529]	0.96
Log IL-8	DHA	Overall: 0.024 [0.002, 0.045]	0.04
		Healthy: 0.017 [−0.028, 0.061]	0.46
		Non-healthy: 0.025 [0.003, 0.047]	0.03
	Omega-6	Overall: −0.26 [−0.76, 0.24]	0.31
		Healthy: −0.16 [−0.006, 0.566]	0.67
		Non-healthy: −0.353 [−1.003, 0.296]	0.29
Log IL-6	DHA	Overall: 0.003 [−0.006, 0.012]	0.49
		Healthy: −0.002 [−0.016, 0.012]	0.76
		Non-healthy: 0.008 [−0.003, 0.018]	0.17
	Omega-3	Overall: 0.043 [0.002, 0.083]	0.04
		Healthy: 0.071 [0.01, 0.133]	0.02
		Non-healthy: 0.028 [−0.022, 0.077]	0.27

CRP: C-reactive protein; Healthy = “Healthy Group 1” (no chorioamnionitis, normal body mass index); IL: interleukin.

## Discussion

This study provides evidence that the pro-inflammatory state associated with pre- and peri-pregnancy ow/ob, as well as other inflammatory states, such as pre-eclampsia, are associated with MOM with temporarily higher markers of inflammation. Overweight and obesity are known to be pro-inflammatory, as well as associated with many co-morbidities, including gestational diabetes, hypertension, and pre-eclampsia, which in and of themselves are pro-inflammatory (4).

### Impact of inflammation on MOM

Although MOM is the gold standard for feeding preterm infants, before this study, data were lacking as to whether the inflammatory markers from maternal inflammatory states were passed to infants through preterm MOM, such as has been reported in term infants whose ow/ob mothers produced MOM with a pro-inflammatory FA profile (11, 12). This is potentially important because, in the vulnerable preterm population, increased inflammation, in general, is associated with conditions such as increased bronchopulmonary dysplasia and poorer long-term outcomes (4). Although there were some minor differences at baseline and in rates of change over time that we detailed, overall MOM from “inflamed” mothers was quite similar to those with fewer inflammatory diagnoses, at least from a cytokine and FA standpoint. Differences in the baseline for IL-8 interestingly showed lower levels in mothers experiencing inflammatory states as well as a positive correlation with DHA, contrary to previous work in a pre-eclamptic term population by Erbağcı et al. (20). This provides some early evidence that MOM from mothers who gave birth while experiencing inflammatory states is relatively similar to that of healthier mothers, especially after the first few weeks postpartum, providing reassurance to mothers who worry they might make “inferior” MOM.

### C-reactive protein

Higher levels of CRP demonstrated in MOM samples from mothers experiencing inflammatory states than less-inflamed, normal-weight mothers suggests that the inflammation affecting the fetal environment could continue to impact the preterm infant after birth through MOM. The significant difference in CRP was demonstrated at baseline (day 5), and CRP trajectory did not differ between most groups. Therefore, although all CRP levels decreased over time, obese/overweight mothers continued to make milk with higher CRP, similar to Whitaker et al. who showed MOM CRP to be positively associated with maternal BMI at 1 month postpartum (21). In addition, we found a negative association between CRP and DHA levels in healthy mothers but not in the mothers experiencing inflammatory states. Perhaps, there are cumulative impacts of inflammation from birth (i.e., PTL and cesarian section) on top of the pro-inflammatory pregnancy states (ow/ob, pre-eclampsia, diabetes, etc.), which lead to higher levels of CRP in the MOM of more inflamed mothers at baseline and although this effect dissipates somewhat over time postpartum, at-risk mothers never reach the levels of their non-inflamed counterparts. In theory, these differences in MOM could result in “intergenerational transmission of disease risk” (21). Reassuringly, however, unlike in previous studies of term infants, there were few significant differences between any other inflammatory markers in MOM between inflamed and non-inflamed groups of mothers, including IL-6, which is a precursor to CRP. This lack of difference in MOM IL-6 by maternal BMI has previously been reported in mothers of term infants (21). It is still unclear whether the short-term period of raised levels of CRP in MOM could have a clinically significant impact on preterm infant health. CRP itself is an indirect measure, not a cause, of inflammation, and there is no evidence of which we are aware that it would be absorbed by the infant, but it could, in theory, bind to infant gastrointestinal tissues with unclear effect. Interestingly, MOM choline, which previously has been reported to be inversely correlated to MOM CRP, did not show similar associations (13) in our cohort. Overall, the lack of disparities in more direct measures of inflammation, such as cytokines, is reassuring. It is likely that the robust benefits of MOM far outweigh any potential risks, especially given that formula is well known to lead to a higher risk of NEC and donor milk lacks many of the benefits of MOM (10).

### Fatty acid profiles

There were some differences in FA levels between groups. Healthier normal-weight mothers had more favorable FA profiles, including lower levels of saturated fat and higher MUFA levels at baseline and a DHA trajectory that increased in the first weeks postpartum. These findings are similar to a previous small study of mothers of preterm infants by Robinson et al., although 40% of mothers in that study were taking DHA supplements (22). We did not have data on DHA supplementation or nutritional intake in our cohort. In addition, grouping overweight and obese mothers together, as BMI and potential inflammation is a spectrum, could affect our results, as previous analyses from a larger number of mothers from this cohort reported that it was specifically the obese mothers who had lower MOM DHA levels (18). Studies have shown that maternal dietary intake has a significant impact on FA composition in MOM, particularly dietary fat and FA composition (23, 24). There could be potential to optimize MOM in at-risk populations by increasing dietary DHA, for example, which could potentially counteract some of the FA differences seen in the “inflamed” MOM and perhaps improve infant outcomes (25–27).

### Clinical significance

The significance of these results is multi-fold. Globally, there continues to be an increase in pre-pregnancy and peri-pregnancy ow/ob. This study, although small, adds to the very limited body of work on inflammatory markers in preterm MOM and suggests the need for further research on the potential impacts of ow/ob on MOM and its subsequent effects on the next generation, especially in vulnerable preterm populations. DHA and choline, for example, are likely important in fetal and infant brain and eye development (27); therefore, suboptimal MOM levels could have long-term ramifications. Work in term populations suggests MOM cytokines like IL-6 and TNFα can correlate with infant adiposity (28). As preterm delivery and pregnancy complications/inflammatory states are more common in racialized groups, research is an important area of advocacy, particularly to mitigate inequities in minority populations (1, 2).

### Study limitations

This study’s limitations include the relatively small sample size, particularly at later time points when fewer leftover MOM samples were available for analysis, the lack of maternal dietary records or information on nutritional supplementation (such as DHA), and the lack of infant outcome data. There is a need for future studies to prospectively evaluate the profile of inflammatory markers and FAs in MOM of preterm infants born to mothers with and without inflammatory states, controlling for dietary intake (especially the FA profile of the diet). Future studies should include short-term outcome data, such as incidence of necrotizing enterocolitis, bronchopulmonary dysplasia, and sepsis, and long-term outcome data, including growth and neurodevelopment. Because of the exploratory nature of this study and relatively small sample size, there was a large number of associations examined and no correction for multiple comparisons, which increases the risk of Type 1 error. Future research testing is needed to test the hypotheses raised by this exploratory analysis. In addition, while healthy groupings examined in this study are clinically relevant to this population, it is possible that other differences between the groups not measured or adjusted for in the present work may be driving some relationships.

## Conclusion

In summary, in the early postpartum days (day 5), we report for the first time that inflammatory states in mothers are associated with higher CRP in MOM. As rates of change did not differ over time, ow/ob status was associated with MOM with higher CRP levels throughout the study. Reassuringly, there were few other associations between most other cytokines/inflammatory markers or FA profiles and maternal inflammatory states; however, we found some suggestions of a more ideal FA profile in mothers with fewer inflammatory diagnoses. Prospective studies are needed to further investigate the inflammatory profile of MOM from mothers experiencing inflammatory states, including ow/ob who deliver preterm infants, potential long-term impacts to vulnerable preterm infants, and how or if MOM can be optimized.

## Data availability statement

The datasets presented in this article are not readily available because this analysis involved secondary use of data from a previous study (for which primary and senior authors were not PIs). Requests to access the datasets should be directed to rebecca.hoban@seattlechildrens.org.

## Ethics statement

The studies involving humans were approved by Mount Sinai Health System (Toronto, Canada) and The Hospital for Sick Children (Toronto, Canada). The studies were conducted in accordance with the local legislation and institutional requirements. Written informed consent for participation in this study was provided by the participants’ legal guardians/next of kin.

## Author contributions

EL-C: Data curation, Writing – original draft, Writing – review & editing. DO’C: Data curation, Funding acquisition, Resources, Writing – review & editing. SU: Data curation, Funding acquisition, Resources, Writing – review & editing, Conceptualization. KH: Data curation, Investigation, Validation, Writing – review & editing. ES: Formal analysis, Methodology, Software, Visualization, Writing – original draft, Writing – review & editing. HN: Data curation, Investigation, Writing – review & editing. RH: Conceptualization, Data curation, Funding acquisition, Investigation, Methodology, Project administration, Resources, Supervision, Visualization, Writing – original draft, Writing – review & editing.
