# Supplementary figures and images for: Associations of maternal inflammatory states with human milk composition in mothers of preterm infants
Source: Front Nutr. 2024 Feb 2;10:1290690. doi: 10.3389/fnut.2023.1290690 (PMC11025471; doi:10.3389/fnut.2023.1290690)

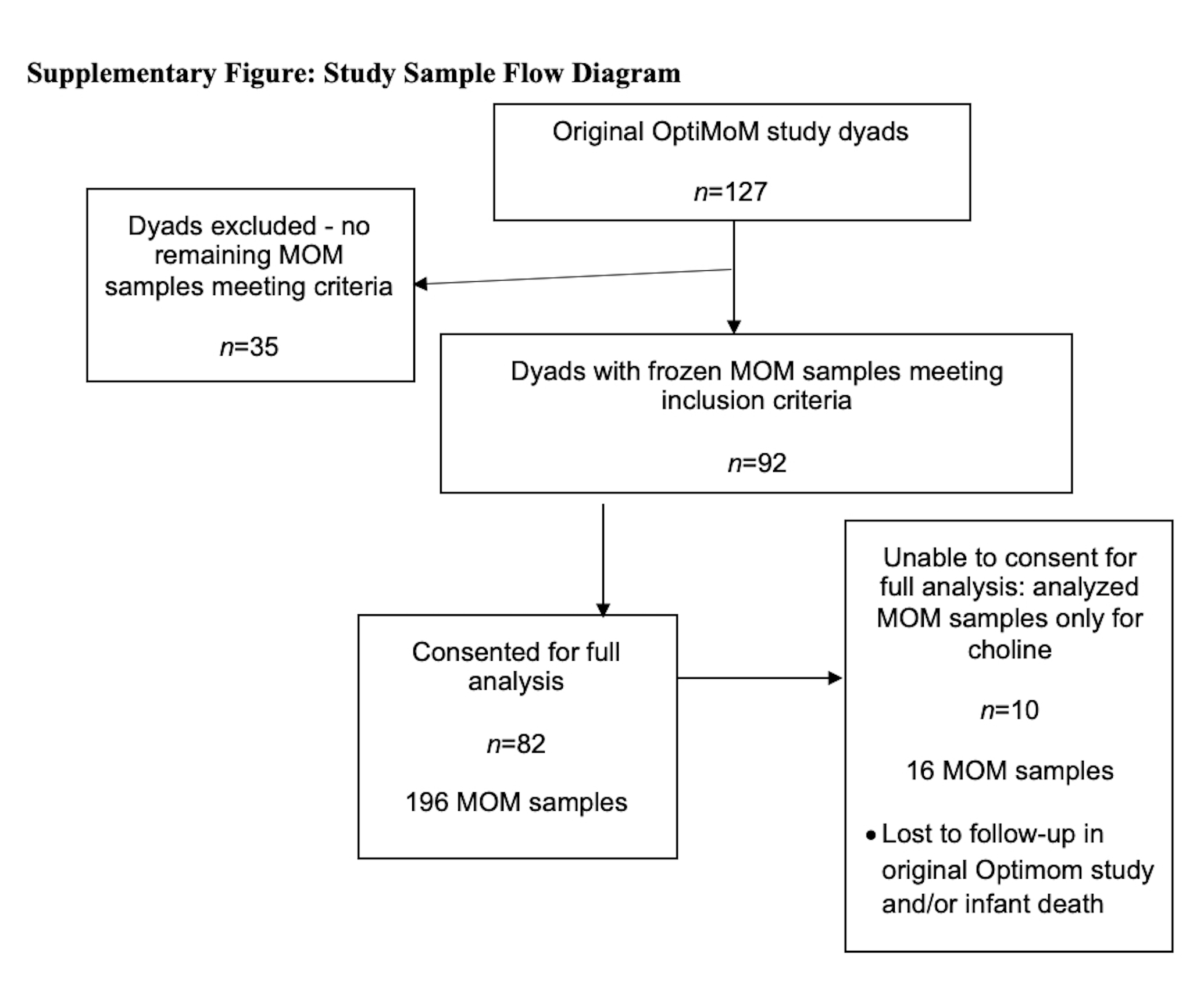

Supplement: Supplementary file 1 [file Image_1.jpg]
