# Supplementary material for: Associations of maternal inflammatory states with human milk composition in mothers of preterm infants
Source: Front Nutr. 2024 Feb 2;10:1290690. doi: 10.3389/fnut.2023.1290690 (PMC11025471; doi:10.3389/fnut.2023.1290690)
Supplement: Supplementary file 2 [file Table_1.docx]

Supplementary Table 1: Percent observations below kit detection limit at each collection period.

|  | **Week 1** | **Week 2** | **Week 3** | **Week 4** | **Week 7** | **Week 10** |
| --- | --- | --- | --- | --- | --- | --- |
| IL-1 βeta | 21 (39.6%) | 25 (61.0%) | 14 (43.8%) | 13 (54.2%) | 23 (60.5%) | 6 (75.0%) |
| IL-1ra | 7 (13.2%) | 6 (14.6%) | 5 (15.6%) | 4 (16.7%) | 7 (18.4%) | 0 (0.0%) |
| IL-6 | 19 (35.8%) | 20 (48.8%) | 11 (34.4%) | 10 (41.7%) | 17 (44.7%) | 4 (50.0%) |
| IL-8 | 0 (0%) | 0 (0%) | 0 (0%) | 0 (0%) | 0 (0%) | 0 (0%) |
| IL-10 | 29 (54.7%) | 25 (61.0%) | 18 (56.2%) | 13 (54.2%) | 23 (60.5%) | 6 (75.0%) |
| IFN-gamma | 33 (62.3%) | 21 (51.2%) | 15 (46.9%) | 9 (37.5%) | 15 (39.5%) | 4 (50.0%) |
| TNF-alpha | 5 (9.4%) | 4 (9.8%) | 3 (9.4%) | 0 (0.0%) | 2 (5.3%) | 1 (12.5%) |
| C-reactive protein (CRP) | 0 (0%) ^a^ | 0 (0%) | 0 (0%) | 0 (0%) | 0 (0%) | 0 (0%) |
| Free choline | 0 (0.0%) ^b^ | 0 (0.0%) | 0 (0.0%) | 1 (3.7%) | 1 (2.5%) | 2 (22.2%) |

^a^ n=2 (3.8%) CRP above detection limit; ^b^ n=1 (1.6%) free choline above detection limit
