# Supplementary material for: Associations of maternal inflammatory states with human milk composition in mothers of preterm infants
Source: Front Nutr. 2024 Feb 2;10:1290690. doi: 10.3389/fnut.2023.1290690 (PMC11025471; doi:10.3389/fnut.2023.1290690)
Supplement: Supplementary file 3 [file Table_2.docx]

| **Inflammatory Marker/Fatty Acid**  **Supplemental Table 2: Associations of Inflammatory Markers and Fatty Acids with Inflammatory States** | **Coef [95% CI]** | **p** |
| --- | --- | --- |
| **Log CRP** | | |
| Chorioamnionitis: Baseline | 0.089 [-0.682, 0.859] | 0.82 |
| Chorioamnionitis: Trajectory | - | 0.52 |
| PROM: Baseline | -0.386 [-1.312, 0.541] | 0.41 |
| PROM: Trajectory | - | 0.84 |
| Caesarian delivery: Baseline | 0.738 [-0.05, 1.526] | 0.066 |
| Caesarian delivery: Trajectory | - | 0.29 |
| PTL: Baseline | -0.164 [-0.878, 0.551] | 0.65 |
| PTL: Trajectory | - | 0.39 |
| Pre-eclampsia: Baseline | 0.472 [-0.171, 1.116] | 0.150 |
| Pre-eclampsia: Trajectory | - | 0.46 |
| OW/OB: Baseline | 0.772 [0.187, 1.357] | 0.010 |
| OW/OB: Trajectory | - | 0.38 |
| Healthy Group 1 (no chorio, normal BMI): Baseline | -0.779 [-1.47, -0.089] | 0.027 |
| Healthy Group 1 (no chorio, normal BMI): Trajectory | - | 0.55 |
| Healthy Group 2 (no chorio, normal BMI, no pre-eclampsia): Baseline | -1.122 [-1.824, -0.419] | 0.002 |
| Healthy Group 2 (no chorio, normal BMI, no pre-eclampsia): Trajectory | - | 0.029 |
| **Log Free Choline** | | |
| Chorioamnionitis: Baseline | -0.125 [-0.734, 0.484] | 0.69 |
| Chorioamnionitis: Trajectory | - | 0.107 |
| PROM: Baseline | 0.145 [-0.393, 0.683] | 0.60 |
| PROM: Trajectory | - | 0.187 |
| Caesarian delivery: Baseline | 0.466 [-0.136, 1.068] | 0.129 |
| Caesarian delivery: Trajectory | - | 0.013 |
| PTL: Baseline | -0.261 [-0.743, 0.222] | 0.29 |
| PTL: Trajectory | - | 0.066 |
| Pre-eclampsia: Baseline | -0.077 [-0.516, 0.362] | 0.73 |
| Pre-eclampsia: Trajectory | - | 0.52 |
| OW/OB: Baseline | -0.187 [-0.664, 0.29] | 0.44 |
| OW/OB: Trajectory | - | 0.120 |
| Healthy Group 1 (no chorio, normal BMI): Baseline | 0.24 [-0.246, 0.725] | 0.33 |
| Healthy Group 1 (no chorio, normal BMI): Trajectory | - | 0.118 |
| Healthy Group 2 (no chorio, normal BMI, no pre-eclampsia): Baseline | 0.323 [-0.229, 0.875] | 0.25 |
| Healthy Group 2 (no chorio, normal BMI, no pre-eclampsia): Trajectory | - | 0.048 |
| **Log IFNγ** | | |
| Chorioamnionitis: Baseline | 0.356 [-2.641, 3.354] | 0.82 |
| Chorioamnionitis: Trajectory | - | 0.91 |
| PROM: Baseline | 0.069 [-2.558, 2.696] | 0.96 |
| PROM: Trajectory | - | 0.94 |
| Caesarian delivery: Baseline | 0.87 [-1.217, 2.957] | 0.41 |
| Caesarian delivery: Trajectory | - | 0.78 |
| PTL: Baseline | -0.48 [-2.65, 1.69] | 0.66 |
| PTL: Trajectory | - | 0.95 |
| Pre-eclampsia: Baseline | 1.164 [-1.501, 3.829] | 0.39 |
| Pre-eclampsia: Trajectory | - | 0.93 |
| OW/OB: Baseline | -0.949 [-2.964, 1.065] | 0.36 |
| OW/OB: Trajectory | - | 0.80 |
| Healthy Group 1 (no chorio, normal BMI): Baseline | 0.52 [-1.775, 2.816] | 0.66 |
| Healthy Group 1 (no chorio, normal BMI): Trajectory | - | 0.98 |
| Healthy Group 2 (no chorio, normal BMI, no pre-eclampsia): Baseline | -0.564 [-3.075, 1.946] | 0.66 |
| Healthy Group 2 (no chorio, normal BMI, no pre-eclampsia): Trajectory | - | 0.96 |
| **Log IL-10** | | |
| Chorioamnionitis: Baseline | -0.912 [-5.064, 3.239] | 0.67 |
| Chorioamnionitis: Trajectory | - | 0.94 |
| PROM: Baseline | -1.116 [-5.323, 3.091] | 0.60 |
| PROM: Trajectory | - | 0.46 |
| Caesarian delivery: Baseline | 1.646 [-2.148, 5.439] | 0.40 |
| Caesarian delivery: Trajectory | - | 0.95 |
| PTL: Baseline | -1.978 [-5.459, 1.502] | 0.27 |
| PTL: Trajectory | - | 0.77 |
| Pre-eclampsia: Baseline | 1.581 [-1.762, 4.923] | 0.35 |
| Pre-eclampsia: Trajectory | - | 0.95 |
| OW/OB: Baseline | -1.065 [-4.431, 2.301] | 0.54 |
| OW/OB: Trajectory | - | 0.99 |
| Healthy Group 1 (no chorio, normal BMI): Baseline | 1.43 [-1.734, 4.594] | 0.38 |
| Healthy Group 1 (no chorio, normal BMI): Trajectory | - | 0.81 |
| Healthy Group 2 (no chorio, normal BMI, no pre-eclampsia): Baseline | 0.058 [-3.498, 3.613] | 0.97 |
| Healthy Group 2 (no chorio, normal BMI, no pre-eclampsia): Trajectory | - | 0.99 |
| **Log IL-1β** | | |
| Chorioamnionitis: Baseline | -0.876 [-3.471, 1.72] | 0.51 |
| Chorioamnionitis: Trajectory | - | 0.95 |
| PROM: Baseline | -0.159 [-2.696, 2.379] | 0.90 |
| PROM: Trajectory | - | 0.92 |
| Caesarian delivery: Baseline | 1.243 [-1.201, 3.688] | 0.32 |
| Caesarian delivery: Trajectory | - | 0.83 |
| PTL: Baseline | -1.137 [-3.199, 0.925] | 0.28 |
| PTL: Trajectory | - | 0.41 |
| Pre-eclampsia: Baseline | 0.907 [-1.711, 3.525] | 0.50 |
| Pre-eclampsia: Trajectory | - | 0.45 |
| OW/OB: Baseline | -0.562 [-2.636, 1.511] | 0.59 |
| OW/OB: Trajectory | - | 0.80 |
| Healthy Group 1 (no chorio, normal BMI): Baseline | 0.973 [-1.292, 3.239] | 0.40 |
| Healthy Group 1 (no chorio, normal BMI): Trajectory | - | 0.70 |
| Healthy Group 2 (no chorio, normal BMI, no pre-eclampsia): Baseline | 0.402 [-1.929, 2.734] | 0.74 |
| Healthy Group 2 (no chorio, normal BMI, no pre-eclampsia): Trajectory | - | 0.63 |
| **Log IL-1ra** | | |
| Chorioamnionitis: Baseline | -1.246 [-3.693, 1.201] | 0.32 |
| Chorioamnionitis: Trajectory | - | 0.55 |
| PROM: Baseline | 0.529 [-1.858, 2.916] | 0.66 |
| PROM: Trajectory | - | 0.60 |
| Caesarian delivery: Baseline | 0.64 [-1.855, 3.134] | 0.62 |
| Caesarian delivery: Trajectory | - | 0.95 |
| PTL: Baseline | -0.408 [-2.674, 1.858] | 0.72 |
| PTL: Trajectory | - | 0.86 |
| Pre-eclampsia: Baseline | -0.261 [-3.081, 2.558] | 0.86 |
| Pre-eclampsia: Trajectory | - | 0.62 |
| OW/OB: Baseline | 0.148 [-2.383, 2.679] | 0.91 |
| OW/OB: Trajectory | - | 0.76 |
| Healthy Group 1 (no chorio, normal BMI): Baseline | 0.735 [-1.446, 2.916] | 0.51 |
| Healthy Group 1 (no chorio, normal BMI): Trajectory | - | 0.77 |
| Healthy Group 2 (no chorio, normal BMI, no pre-eclampsia): Baseline | 0.599 [-1.569, 2.767] | 0.59 |
| Healthy Group 2 (no chorio, normal BMI, no pre-eclampsia): Trajectory | - | 0.63 |
| **Log IL-6** | | |
| Chorioamnionitis: Baseline | -0.523 [-3.691, 2.646] | 0.75 |
| Chorioamnionitis: Trajectory | - | 0.78 |
| PROM: Baseline | -0.184 [-3.599, 3.231] | 0.92 |
| PROM: Trajectory | - | 0.37 |
| Caesarian delivery: Baseline | 0.051 [-3.098, 3.2] | 0.97 |
| Caesarian delivery: Trajectory | - | 0.75 |
| PTL: Baseline | -1.193 [-3.923, 1.536] | 0.39 |
| PTL: Trajectory | - | 0.70 |
| Pre-eclampsia: Baseline | 1.228 [-1.781, 4.236] | 0.42 |
| Pre-eclampsia: Trajectory | - | 0.29 |
| OW/OB: Baseline | 0.488 [-2.369, 3.345] | 0.74 |
| OW/OB: Trajectory | - | 0.59 |
| Healthy Group 1 (no chorio, normal BMI): Baseline | 0.058 [-2.778, 2.894] | 0.97 |
| Healthy Group 1 (no chorio, normal BMI): Trajectory | - | 0.91 |
| Healthy Group 2 (no chorio, normal BMI, no pre-eclampsia): Baseline | -0.983 [-4.166, 2.2] | 0.55 |
| Healthy Group 2 (no chorio, normal BMI, no pre-eclampsia): Trajectory | - | 0.39 |
| **Log IL-8** | | |
| Chorioamnionitis: Baseline | -1.187 [-2.29, -0.084] | 0.035 |
| Chorioamnionitis: Trajectory | - | 0.64 |
| PROM: Baseline | -0.518 [-1.698, 0.663] | 0.39 |
| PROM: Trajectory | - | 0.98 |
| Caesarian delivery: Baseline | 0.957 [-0.089, 2.003] | 0.073 |
| Caesarian delivery: Trajectory | - | 0.71 |
| PTL: Baseline | -1.002 [-1.983, -0.021] | 0.045 |
| PTL: Trajectory | - | 0.59 |
| Pre-eclampsia: Baseline | 0.372 [-0.658, 1.401] | 0.48 |
| Pre-eclampsia: Trajectory | - | 0.68 |
| OW/OB: Baseline | 0.234 [-0.784, 1.253] | 0.65 |
| OW/OB: Trajectory | - | 0.79 |
| Healthy Group 1 (no chorio, normal BMI): Baseline | 0.629 [-0.353, 1.611] | 0.21 |
| Healthy Group 1 (no chorio, normal BMI): Trajectory | - | 0.75 |
| Healthy Group 2 (no chorio, normal BMI, no pre-eclampsia): Baseline | 0.394 [-0.728, 1.515] | 0.49 |
| Healthy Group 2 (no chorio, normal BMI, no pre-eclampsia): Trajectory | - | 0.119 |
| **Log TNFα** | | |
| Chorioamnionitis: Baseline | -0.165 [-1.496, 1.166] | 0.81 |
| Chorioamnionitis: Trajectory | - | 0.74 |
| PROM: Baseline | -0.844 [-2.847, 1.159] | 0.41 |
| PROM: Trajectory | - | 0.23 |
| Caesarian delivery: Baseline | 1.282 [-0.037, 2.601] | 0.057 |
| Caesarian delivery: Trajectory | - | 0.071 |
| PTL: Baseline | -0.256 [-1.408, 0.896] | 0.66 |
| PTL: Trajectory | - | 0.37 |
| Pre-eclampsia: Baseline | 1.096 [-0.157, 2.349] | 0.086 |
| Pre-eclampsia: Trajectory | - | 0.198 |
| OW/OB: Baseline | -0.821 [-2.086, 0.444] | 0.20 |
| OW/OB: Trajectory | - | 0.30 |
| Healthy Group 1 (no chorio, normal BMI): Baseline | 0.618 [-0.499, 1.734] | 0.28 |
| Healthy Group 1 (no chorio, normal BMI): Trajectory | - | 0.43 |
| Healthy Group 2 (no chorio, normal BMI, no pre-eclampsia): Baseline | 0.189 [-1.036, 1.414] | 0.76 |
| Healthy Group 2 (no chorio, normal BMI, no pre-eclampsia): Trajectory | - | 0.91 |
| **Omega-6 fatty acids** | | |
| Chorioamnionitis: Baseline | -0.03 [-0.052, -0.007] | 0.010 |
| Chorioamnionitis: Trajectory | - | 0.33 |
| PROM: Baseline | -0.009 [-0.032, 0.014] | 0.47 |
| PROM: Trajectory | - | 0.52 |
| Caesarian delivery: Baseline | 0.002 [-0.028, 0.033] | 0.87 |
| Caesarian delivery: Trajectory | - | 0.93 |
| PTL: Baseline | -0.007 [-0.031, 0.018] | 0.60 |
| PTL: Trajectory | - | 0.68 |
| Pre-eclampsia: Baseline | 0.004 [-0.022, 0.031] | 0.74 |
| Pre-eclampsia: Trajectory | - | 0.82 |
| OW/OB: Baseline | 0.001 [-0.025, 0.026] | 0.97 |
| OW/OB: Trajectory | - | 0.077 |
| Healthy Group 1 (no chorio, normal BMI): Baseline | 0.021 [-0.004, 0.045] | 0.094 |
| Healthy Group 1 (no chorio, normal BMI): Trajectory | - | 0.26 |
| Healthy Group 2 (no chorio, normal BMI, no pre-eclampsia): Baseline | 0.016 [-0.009, 0.042] | 0.20 |
| Healthy Group 2 (no chorio, normal BMI, no pre-eclampsia): Trajectory | - | 0.181 |
| **Omega-3 fatty acids** | | |
| Chorioamnionitis: Baseline | -0.004 [-0.011, 0.002] | 0.181 |
| Chorioamnionitis: Trajectory | - | 0.54 |
| PROM: Baseline | -0.002 [-0.009, 0.006] | 0.67 |
| PROM: Trajectory | - | 0.60 |
| Caesarian delivery: Baseline | -0.001 [-0.008, 0.006] | 0.79 |
| Caesarian delivery: Trajectory | - | 0.90 |
| PTL: Baseline | -0.003 [-0.009, 0.003] | 0.34 |
| PTL: Trajectory | - | 0.66 |
| Pre-eclampsia: Baseline | 0 [-0.005, 0.005] | 0.95 |
| Pre-eclampsia: Trajectory | - | 0.66 |
| OW/OB: Baseline | 0.004 [-0.002, 0.009] | 0.21 |
| OW/OB: Trajectory | - | 0.31 |
| Healthy Group 1 (no chorio, normal BMI): Baseline | 0.001 [-0.005, 0.007] | 0.73 |
| Healthy Group 1 (no chorio, normal BMI): Trajectory | - | 0.74 |
| Healthy Group 2 (no chorio, normal BMI, no pre-eclampsia): Baseline | 0 [-0.007, 0.007] | 0.92 |
| Healthy Group 2 (no chorio, normal BMI, no pre-eclampsia): Trajectory | - | 0.64 |
| **Omega-6:Omega-3 Ratio** | | |
| Chorioamnionitis: Baseline | 1.375 [-3.983, 6.733] | 0.61 |
| Chorioamnionitis: Trajectory | - | 0.035 |
| PROM: Baseline | 2.976 [-4.41, 10.363] | 0.43 |
| PROM: Trajectory | - | 0.74 |
| Caesarian delivery: Baseline | 1.615 [-1.077, 4.308] | 0.24 |
| Caesarian delivery: Trajectory | - | 0.49 |
| PTL: Baseline | 1.657 [-1.897, 5.212] | 0.36 |
| PTL: Trajectory | - | 0.86 |
| Pre-eclampsia: Baseline | -1.005 [-3.459, 1.448] | 0.42 |
| Pre-eclampsia: Trajectory | - | 0.90 |
| OW/OB: Baseline | -2.261 [-4.942, 0.419] | 0.098 |
| OW/OB: Trajectory | - | 0.018 |
| Healthy Group 1 (no chorio, normal BMI): Baseline | 0.648 [-2.649, 3.944] | 0.70 |
| Healthy Group 1 (no chorio, normal BMI): Trajectory | - | 0.34 |
| Healthy Group 2 (no chorio, normal BMI, no pre-eclampsia): Baseline | 1.421 [-2.184, 5.026] | 0.44 |
| Healthy Group 2 (no chorio, normal BMI, no pre-eclampsia): Trajectory | - | 0.52 |
| **Arachidonic Acid** | | |
| Chorioamnionitis: Baseline | 0 [-0.002, 0.001] | 0.60 |
| Chorioamnionitis: Trajectory | - | 0.26 |
| PROM: Baseline | 0 [-0.001, 0.002] | 0.77 |
| PROM: Trajectory | - | 0.32 |
| Caesarian delivery: Baseline | 0 [-0.001, 0.002] | 0.54 |
| Caesarian delivery: Trajectory | - | 0.42 |
| PTL: Baseline | -0.001 [-0.002, 0.001] | 0.28 |
| PTL: Trajectory | - | 0.063 |
| Pre-eclampsia: Baseline | 0.001 [-0.001, 0.002] | 0.48 |
| Pre-eclampsia: Trajectory | - | 0.086 |
| OW/OB: Baseline | 0 [-0.001, 0.002] | 0.68 |
| OW/OB: Trajectory | - | 0.24 |
| Healthy Group 1 (no chorio, normal BMI): Baseline | 0 [-0.001, 0.002] | 0.80 |
| Healthy Group 1 (no chorio, normal BMI): Trajectory | - | 0.82 |
| Healthy Group 2 (no chorio, normal BMI, no pre-eclampsia): Baseline | -0.001 [-0.002, 0.001] | 0.35 |
| Healthy Group 2 (no chorio, normal BMI, no pre-eclampsia): Trajectory | - | 0.102 |
| **Docosahexaenoic Acid (DHA)** | | |
| Chorioamnionitis: Baseline | -0.001 [-0.002, 0] | 0.191 |
| Chorioamnionitis: Trajectory | - | 0.69 |
| PROM: Baseline | 0 [-0.002, 0.003] | 0.73 |
| PROM: Trajectory | - | 0.88 |
| Caesarian delivery: Baseline | 0 [-0.002, 0.001] | 0.65 |
| Caesarian delivery: Trajectory | - | 0.68 |
| PTL: Baseline | 0 [-0.002, 0.001] | 0.69 |
| PTL: Trajectory | - | 0.31 |
| Pre-eclampsia: Baseline | 0 [-0.002, 0.001] | 0.95 |
| Pre-eclampsia: Trajectory | - | 0.70 |
| OW/OB: Baseline | 0.001 [-0.001, 0.002] | 0.36 |
| OW/OB: Trajectory | - | 0.079 |
| Healthy Group 1 (no chorio, normal BMI): Baseline | 0 [-0.001, 0.002] | 0.86 |
| Healthy Group 1 (no chorio, normal BMI): Trajectory | - | 0.21 |
| Healthy Group 2 (no chorio, normal BMI, no pre-eclampsia): Baseline | 0 [-0.002, 0.001] | 0.59 |
| Healthy Group 2 (no chorio, normal BMI, no pre-eclampsia): Trajectory | - | 0.033 |
| **Linoleic Acid** | | |
| Chorioamnionitis: Baseline | -0.026 [-0.048, -0.004] | 0.021 |
| Chorioamnionitis: Trajectory | - | 0.39 |
| PROM: Baseline | -0.007 [-0.029, 0.016] | 0.56 |
| PROM: Trajectory | - | 0.52 |
| Caesarian delivery: Baseline | 0.002 [-0.028, 0.032] | 0.89 |
| Caesarian delivery: Trajectory | - | 0.93 |
| PTL: Baseline | -0.003 [-0.026, 0.02] | 0.78 |
| PTL: Trajectory | - | 0.69 |
| Pre-eclampsia: Baseline | 0.003 [-0.022, 0.029] | 0.80 |
| Pre-eclampsia: Trajectory | - | 0.74 |
| OW/OB: Baseline | 0 [-0.025, 0.024] | 0.98 |
| OW/OB: Trajectory | - | 0.059 |
| Healthy Group 1 (no chorio, normal BMI): Baseline | 0.019 [-0.004, 0.042] | 0.103 |
| Healthy Group 1 (no chorio, normal BMI): Trajectory | - | 0.23 |
| Healthy Group 2 (no chorio, normal BMI, no pre-eclampsia): Baseline | 0.016 [-0.007, 0.039] | 0.166 |
| Healthy Group 2 (no chorio, normal BMI, no pre-eclampsia): Trajectory | - | 0.106 |
| **Monounsaturated Fatty Acids (MUFA)** | | |
| Chorioamnionitis: Baseline | -0.008 [-0.047, 0.03] | 0.68 |
| Chorioamnionitis: Trajectory | - | 0.114 |
| PROM: Baseline | -0.025 [-0.06, 0.011] | 0.175 |
| PROM: Trajectory | - | 0.051 |
| Caesarian delivery: Baseline | 0.017 [-0.025, 0.058] | 0.43 |
| Caesarian delivery: Trajectory | - | 0.185 |
| PTL: Baseline | -0.005 [-0.039, 0.03] | 0.78 |
| PTL: Trajectory | - | 0.67 |
| Pre-eclampsia: Baseline | 0.015 [-0.019, 0.049] | 0.39 |
| Pre-eclampsia: Trajectory | - | 0.190 |
| OW/OB: Baseline | -0.034 [-0.069, 0.002] | 0.061 |
| OW/OB: Trajectory | - | 0.51 |
| Healthy Group 1 (no chorio, normal BMI): Baseline | 0.02 [-0.012, 0.053] | 0.21 |
| Healthy Group 1 (no chorio, normal BMI): Trajectory | - | 0.48 |
| Healthy Group 2 (no chorio, normal BMI, no pre-eclampsia): Baseline | 0.016 [-0.018, 0.049] | 0.35 |
| Healthy Group 2 (no chorio, normal BMI, no pre-eclampsia): Trajectory | - | 0.95 |
| **Saturated fatty acids** | | |
| Chorioamnionitis: Baseline | 0.041 [0.001, 0.08] | 0.042 |
| Chorioamnionitis: Trajectory | - | 0.36 |
| PROM: Baseline | 0.021 [-0.023, 0.065] | 0.34 |
| PROM: Trajectory | - | 0.101 |
| Caesarian delivery: Baseline | -0.019 [-0.06, 0.022] | 0.37 |
| Caesarian delivery: Trajectory | - | 0.59 |
| PTL: Baseline | 0.009 [-0.029, 0.047] | 0.64 |
| PTL: Trajectory | - | 0.41 |
| Pre-eclampsia: Baseline | -0.017 [-0.052, 0.017] | 0.32 |
| Pre-eclampsia: Trajectory | - | 0.22 |
| OW/OB: Baseline | 0.033 [-0.009, 0.074] | 0.125 |
| OW/OB: Trajectory | - | 0.51 |
| Healthy Group 1 (no chorio, normal BMI): Baseline | -0.042 [-0.077, -0.008] | 0.017 |
| Healthy Group 1 (no chorio, normal BMI): Trajectory | - | 0.122 |
| Healthy Group 2 (no chorio, normal BMI, no pre-eclampsia): Baseline | -0.034 [-0.07, 0.002] | 0.063 |
| Healthy Group 2 (no chorio, normal BMI, no pre-eclampsia): Trajectory | - | 0.120 |

Note: PROM= premature rupture of membranes; PTL=preterm labor; OW/OB=overweight/obese, chorio=chorioamnionitis; BMI=body mass index
