# Supplementary material for: Associations of maternal inflammatory states with human milk composition in mothers of preterm infants
Source: Front Nutr. 2024 Feb 2;10:1290690. doi: 10.3389/fnut.2023.1290690 (PMC11025471; doi:10.3389/fnut.2023.1290690)
Supplement: Supplementary file 4 [file Table_3.docx]

| **Supplemental Table 3: Associations between Inflammatory Markers and Fatty Acids** | | **Overall** | | **Healthy (no chorioamnionitis, normal BMI)** | | **Not healthy** | |
| --- | --- | --- | --- | --- | --- | --- | --- |
| **Predictor** | **Outcome** | **Coef [95% CI]** | **p** | **Coef [95% CI]** | **p** | **Coef [95% CI]** | **p** |
| Log CRP | Arachidonic Acid | 0.014 [-0.013, 0.041] | 0.30 | 0.028 [-0.014, 0.071] | 0.193 | 0.015 [-0.022, 0.052] | 0.43 |
| Log CRP | DHA | -0.023 [-0.046, 0.001] | 0.056 | -0.031 [-0.06, -0.002] | 0.034 | -0.005 [-0.037, 0.026] | 0.74 |
| Log CRP | Linoleic Acid | 0.23 [-0.17, 0.63] | 0.26 | 0.688 [0.009, 1.367] | 0.047 | 0.005 [-0.465, 0.474] | 0.98 |
| Log CRP | MUFA | -0.509 [-1.152, 0.133] | 0.120 | -0.375 [-1.263, 0.514] | 0.41 | -0.21 [-1.124, 0.704] | 0.65 |
| Log CRP | Omega-6 | 0.247 [-0.173, 0.667] | 0.25 | 0.752 [0.053, 1.45] | 0.035 | 0.012 [-0.506, 0.529] | 0.96 |
| Log CRP | Omega-3 | 0.037 [-0.055, 0.129] | 0.43 | 0.066 [-0.081, 0.213] | 0.38 | 0.048 [-0.074, 0.17] | 0.44 |
| Log CRP | Omega6:Omega3 | 0.01 [-0.333, 0.353] | 0.95 | 0.073 [-0.25, 0.395] | 0.66 | -0.11 [-0.628, 0.408] | 0.68 |
| Log CRP | SFA | 0.236 [-0.574, 1.046] | 0.57 | -0.442 [-1.496, 0.612] | 0.41 | 0.205 [-0.862, 1.271] | 0.71 |
| Log Free Choline | Arachidonic Acid | 0.021 [-0.02, 0.062] | 0.31 | -0.005 [-0.079, 0.069] | 0.89 | 0.036 [-0.014, 0.086] | 0.163 |
| Log Free Choline | DHA | -0.003 [-0.05, 0.043] | 0.89 | -0.045 [-0.116, 0.027] | 0.22 | 0.023 [-0.035, 0.082] | 0.43 |
| Log Free Choline | Linoleic Acid | -0.173 [-0.754, 0.407] | 0.56 | -0.25 [-1.383, 0.882] | 0.66 | -0.3 [-0.929, 0.33] | 0.35 |
| Log Free Choline | MUFA | -0.267 [-1.167, 0.632] | 0.56 | -0.629 [-1.768, 0.51] | 0.28 | -0.162 [-1.277, 0.953] | 0.78 |
| Log Free Choline | Omega-6 | -0.1 [-0.729, 0.53] | 0.76 | -0.2 [-1.41, 1.009] | 0.75 | -0.206 [-0.899, 0.486] | 0.56 |
| Log Free Choline | Omega-3 | -0.033 [-0.211, 0.144] | 0.71 | -0.027 [-0.31, 0.256] | 0.85 | -0.028 [-0.248, 0.192] | 0.80 |
| Log Free Choline | Omega6:Omega3 | 0.351 [-0.341, 1.044] | 0.32 | 0.265 [-0.89, 1.421] | 0.65 | 0.264 [-0.549, 1.078] | 0.52 |
| Log Free Choline | SFA | 0.249 [-0.964, 1.461] | 0.69 | 0.63 [-0.749, 2.01] | 0.37 | 0.284 [-1.251, 1.818] | 0.72 |
| Log IFN-Ɣ | Arachidonic Acid | 0.002 [-0.011, 0.015] | 0.76 | 0.003 [-0.018, 0.024] | 0.78 | -0.001 [-0.019, 0.017] | 0.91 |
| Log IFN-Ɣ | DHA | 0.003 [-0.01, 0.015] | 0.67 | 0 [-0.019, 0.019] | 0.99 | 0.001 [-0.015, 0.016] | 0.90 |
| Log IFN-Ɣ | Linoleic Acid | 0.158 [-0.071, 0.387] | 0.176 | 0.052 [-0.302, 0.406] | 0.77 | 0.207 [-0.081, 0.495] | 0.159 |
| Log IFN-Ɣ | MUFA | 0.25 [-0.1, 0.6] | 0.161 | -0.072 [-0.536, 0.393] | 0.76 | 0.39 [-0.088, 0.868] | 0.110 |
| Log IFN-Ɣ | Omega-6 | 0.142 [-0.099, 0.384] | 0.25 | 0.037 [-0.338, 0.412] | 0.85 | 0.187 [-0.117, 0.491] | 0.23 |
| Log IFN-Ɣ | Omega-3 | 0.065 [0.018, 0.112] | 0.007 | 0.08 [-0.002, 0.163] | 0.057 | 0.05 [-0.009, 0.109] | 0.095 |
| Log IFN-Ɣ | Omega6:Omega3 | -0.198 [-0.4, 0.003] | 0.054 | -0.328 [-0.665, 0.009] | 0.056 | -0.105 [-0.347, 0.137] | 0.39 |
| Log IFN-Ɣ | SFA | -0.475 [-0.892, -0.058] | 0.025 | -0.09 [-0.617, 0.436] | 0.74 | -0.625 [-1.182, -0.068] | 0.028 |
| Log IL-10 | Arachidonic Acid | 0.007 [-0.003, 0.016] | 0.160 | 0.007 [-0.008, 0.021] | 0.37 | 0.005 [-0.007, 0.018] | 0.39 |
| Log IL-10 | DHA | 0.006 [-0.003, 0.016] | 0.178 | 0.001 [-0.013, 0.014] | 0.91 | 0.007 [-0.004, 0.019] | 0.21 |
| Log IL-10 | Linoleic Acid | -0.072 [-0.231, 0.088] | 0.38 | -0.052 [-0.269, 0.166] | 0.64 | -0.096 [-0.32, 0.127] | 0.40 |
| Log IL-10 | MUFA | 0.156 [-0.062, 0.374] | 0.161 | -0.058 [-0.362, 0.246] | 0.71 | 0.257 [-0.054, 0.567] | 0.106 |
| Log IL-10 | Omega-6 | -0.069 [-0.237, 0.099] | 0.42 | -0.042 [-0.28, 0.197] | 0.73 | -0.102 [-0.334, 0.13] | 0.39 |
| Log IL-10 | Omega-3 | 0.026 [-0.013, 0.064] | 0.197 | 0.044 [-0.012, 0.1] | 0.127 | 0.009 [-0.041, 0.059] | 0.73 |
| Log IL-10 | Omega6:Omega3 | -0.138 [-0.327, 0.052] | 0.154 | -0.165 [-0.354, 0.025] | 0.089 | -0.11 [-0.383, 0.163] | 0.43 |
| Log IL-10 | SFA | -0.132 [-0.418, 0.155] | 0.37 | -0.016 [-0.354, 0.322] | 0.93 | -0.151 [-0.547, 0.245] | 0.46 |
| Log IL-1β | Arachidonic Acid | 0.004 [-0.008, 0.017] | 0.50 | 0.009 [-0.013, 0.031] | 0.41 | 0.002 [-0.013, 0.016] | 0.84 |
| Log IL-1β | DHA | 0.002 [-0.009, 0.014] | 0.68 | -0.003 [-0.023, 0.017] | 0.77 | 0.006 [-0.006, 0.017] | 0.34 |
| Log IL-1β | Linoleic Acid | -0.052 [-0.254, 0.15] | 0.62 | 0.106 [-0.195, 0.407] | 0.49 | -0.158 [-0.42, 0.103] | 0.23 |
| Log IL-1β | MUFA | 0.06 [-0.229, 0.35] | 0.68 | -0.304 [-0.731, 0.123] | 0.163 | 0.222 [-0.156, 0.6] | 0.25 |
| Log IL-1β | Omega-6 | -0.059 [-0.271, 0.153] | 0.59 | 0.116 [-0.209, 0.44] | 0.48 | -0.177 [-0.45, 0.096] | 0.20 |
| Log IL-1β | Omega-3 | 0.04 [-0.01, 0.089] | 0.116 | 0.081 [0.011, 0.152] | 0.023 | 0.018 [-0.042, 0.078] | 0.55 |
| Log IL-1β | Omega6:Omega3 | -0.226 [-0.479, 0.027] | 0.081 | -0.191 [-0.405, 0.022] | 0.078 | -0.264 [-0.633, 0.105] | 0.161 |
| Log IL-1β | SFA | -0.053 [-0.413, 0.308] | 0.77 | 0.016 [-0.42, 0.453] | 0.94 | -0.035 [-0.534, 0.465] | 0.89 |
| Log IL-1ra | Arachidonic Acid | 0.009 [-0.004, 0.021] | 0.190 | 0.003 [-0.018, 0.024] | 0.77 | 0.012 [-0.003, 0.027] | 0.109 |
| Log IL-1ra | DHA | 0.007 [-0.006, 0.019] | 0.28 | 0.002 [-0.02, 0.024] | 0.88 | 0.011 [-0.002, 0.023] | 0.089 |
| Log IL-1ra | Linoleic Acid | -0.023 [-0.237, 0.191] | 0.83 | 0.01 [-0.294, 0.313] | 0.95 | -0.045 [-0.332, 0.242] | 0.76 |
| Log IL-1ra | MUFA | 0.064 [-0.223, 0.352] | 0.66 | -0.208 [-0.543, 0.126] | 0.22 | 0.199 [-0.182, 0.58] | 0.31 |
| Log IL-1ra | Omega-6 | -0.012 [-0.242, 0.218] | 0.92 | 0.028 [-0.304, 0.36] | 0.87 | -0.039 [-0.348, 0.27] | 0.81 |
| Log IL-1ra | Omega-3 | 0.013 [-0.037, 0.063] | 0.60 | 0.038 [-0.054, 0.13] | 0.42 | 0.002 [-0.051, 0.055] | 0.94 |
| Log IL-1ra | Omega6:Omega3 | -0.031 [-0.194, 0.132] | 0.71 | -0.105 [-0.358, 0.149] | 0.42 | 0.004 [-0.189, 0.198] | 0.97 |
| Log IL-1ra | SFA | -0.015 [-0.396, 0.366] | 0.94 | 0.188 [-0.298, 0.673] | 0.45 | -0.114 [-0.606, 0.378] | 0.65 |
| Log IL-6 | Arachidonic Acid | 0 [-0.011, 0.01] | 0.95 | 0.006 [-0.009, 0.022] | 0.43 | -0.004 [-0.017, 0.01] | 0.57 |
| Log IL-6 | DHA | 0.003 [-0.006, 0.012] | 0.49 | -0.002 [-0.016, 0.012] | 0.76 | 0.008 [-0.003, 0.018] | 0.169 |
| Log IL-6 | Linoleic Acid | 0.053 [-0.126, 0.232] | 0.56 | 0.191 [-0.057, 0.44] | 0.131 | -0.032 [-0.268, 0.204] | 0.79 |
| Log IL-6 | MUFA | 0.057 [-0.183, 0.297] | 0.64 | -0.136 [-0.509, 0.238] | 0.48 | 0.172 [-0.14, 0.483] | 0.28 |
| Log IL-6 | Omega-6 | 0.045 [-0.144, 0.235] | 0.64 | 0.206 [-0.061, 0.473] | 0.131 | -0.053 [-0.304, 0.198] | 0.68 |
| Log IL-6 | Omega-3 | 0.043 [0.002, 0.083] | 0.041 | 0.071 [0.01, 0.133] | 0.023 | 0.028 [-0.022, 0.077] | 0.27 |
| Log IL-6 | Omega6:Omega3 | -0.138 [-0.31, 0.035] | 0.118 | -0.175 [-0.411, 0.061] | 0.146 | -0.127 [-0.357, 0.102] | 0.28 |
| Log IL-6 | SFA | -0.15 [-0.445, 0.145] | 0.32 | -0.188 [-0.599, 0.223] | 0.37 | -0.129 [-0.537, 0.278] | 0.53 |
| Log IL-8 | Arachidonic Acid | 0.037 [0.013, 0.061] | 0.003 | 0.028 [-0.013, 0.069] | 0.183 | 0.04 [0.011, 0.069] | 0.007 |
| Log IL-8 | DHA | 0.024 [0.002, 0.045] | 0.035 | 0.017 [-0.028, 0.061] | 0.46 | 0.025 [0.003, 0.047] | 0.029 |
| Log IL-8 | Linoleic Acid | -0.324 [-0.801, 0.152] | 0.182 | -0.239 [-0.934, 0.456] | 0.50 | -0.407 [-1.027, 0.212] | 0.198 |
| Log IL-8 | MUFA | 0.028 [-0.555, 0.612] | 0.92 | 0.187 [-0.938, 1.312] | 0.74 | -0.124 [-0.781, 0.532] | 0.71 |
| Log IL-8 | Omega-6 | -0.26 [-0.76, 0.24] | 0.31 | -0.16 [-0.886, 0.566] | 0.67 | -0.353 [-1.003, 0.296] | 0.29 |
| Log IL-8 | Omega-3 | 0.013 [-0.077, 0.103] | 0.78 | 0.064 [-0.108, 0.235] | 0.47 | -0.014 [-0.111, 0.084] | 0.78 |
| Log IL-8 | Omega6:Omega3 | -0.252 [-0.604, 0.1] | 0.161 | -0.274 [-0.728, 0.181] | 0.24 | -0.253 [-0.686, 0.18] | 0.25 |
| Log IL-8 | SFA | 0.341 [-0.483, 1.165] | 0.42 | -0.011 [-1.553, 1.53] | 0.99 | 0.633 [-0.322, 1.587] | 0.194 |
| Log TNF-α | Arachidonic Acid | 0.029 [0.01, 0.048] | 0.003 | 0.04 [0.01, 0.069] | 0.008 | 0.021 [-0.001, 0.044] | 0.060 |
| Log TNF-α | DHA | 0.004 [-0.018, 0.026] | 0.73 | 0.008 [-0.029, 0.045] | 0.67 | 0 [-0.025, 0.024] | 0.99 |
| Log TNF-α | Linoleic Acid | -0.131 [-0.504, 0.243] | 0.49 | -0.45 [-0.951, 0.05] | 0.078 | -0.061 [-0.549, 0.426] | 0.81 |
| Log TNF-α | MUFA | -0.221 [-0.679, 0.238] | 0.35 | -0.095 [-0.778, 0.588] | 0.79 | -0.433 [-0.958, 0.093] | 0.106 |
| Log TNF-α | Omega-6 | -0.07 [-0.463, 0.324] | 0.73 | -0.382 [-0.895, 0.13] | 0.144 | -0.005 [-0.518, 0.507] | 0.98 |
| Log TNF-α | Omega-3 | -0.011 [-0.096, 0.074] | 0.80 | 0.013 [-0.142, 0.169] | 0.87 | -0.029 [-0.127, 0.068] | 0.56 |
| Log TNF-α | Omega6:Omega3 | -0.028 [-0.29, 0.234] | 0.83 | -0.435 [-1.142, 0.271] | 0.23 | 0.146 [-0.123, 0.415] | 0.29 |
| Log TNF-α | SFA | 0.31 [-0.323, 0.943] | 0.34 | 0.388 [-0.408, 1.184] | 0.34 | 0.519 [-0.198, 1.235] | 0.156 |

Note: CRP = C-reactive protein; DHA= Docosahexanoic Acid; IL = interleukin; INF= interferon; MUFA = monounsaturated fatty acid; SFA = saturated fatty acid; TNF = tumor necrosis factor

As the FAs were multiplied by 100 given differences in units, to interpret the table, for example, an average 1 unit increase in log CRP is associated with a 0.023/100 = 0.00023 decrease in percentage of DHA.
